# Stochastic and deterministic processes shape bioenergy crop microbiomes along a vertical soil niche

**DOI:** 10.1111/1462-2920.16269

**Published:** 2022-11-24

**Authors:** Gian Maria Niccolò Benucci, Pedro Beschoren da Costa, Xinxin Wang, Gregory Bonito

**Affiliations:** ^1^ DOE Great Lakes Bioenergy Research Center Michigan State University East Lansing Michigan USA; ^2^ Department of Plant, Soil, and Microbial Sciences Michigan State University East Lansing Michigan USA; ^3^ Department of Microbiology & Molecular Genetics Michigan State University East Lansing USA

## Abstract

Sustainable biofuel cropping systems aim to address climate change while meeting energy needs. Understanding how soil and plant‐associated microbes respond to these different cropping systems is key to promoting agriculture sustainability and evaluating changes in ecosystem functions. Here, we leverage a long‐term biofuel cropping system field experiment to dissect soil and root microbiome changes across a soil‐depth gradient in poplar, restored prairie and switchgrass to understand their effects on the microbial communities. High throughput amplicon sequencing of the fungal internal transcribed spacer (ITS) and prokaryotic 16S DNA regions showed a common trend of root and soil microbial community richness decreasing and evenness increasing with depth. Ecological niche (root vs. soil) had the strongest effect on community structure, followed by depth, then crop. Stochastic processes dominated the structuring of fungal communities in deeper soil layers while operational taxonomic units (OTUs) in surface soil layers were more likely to co‐occur and to be enriched by plant hosts. Prokaryotic communities were dispersal limited at deeper depths. Microbial networks showed a higher density, connectedness, average degree and module size in deeper soils. We observed a decrease in fungal‐fungal links and an increase of bacteria–bacteria links with increasing depth in all crops, particularly in the root microbiome.

## INTRODUCTION

Plants are rich microbial ecosystems and important ecological engineers (Bulgarelli et al., 2013; Delgado‐Baquerizo et al., 2018; Tedersoo et al., 2014). These sessile organisms are anchored to the soil by their roots, which also assist in provisioning water, nutrients and minerals to plants. Root and aboveground plant tissues are populated by a rich diversity of microorganisms known as the plant microbiome. Plant microbiomes are capable of modulating plant health, growth, and development, and have been implicated in crop productivity and ecosystem functioning (Agler et al., 2016; Durán et al., 2018; Howe et al., 2021; Mendes et al., 2013; van der Heijden et al., 2016).

Soils are the largest and most diverse reservoir of microorganisms on the planet (Bickel & Or, 2020; Fierer, 2017). Soil food webs are fuelled by autotrophic metabolism, thus, aboveground plant photosynthesis is critical to soil development. Similarly, the activities of soil microbes that feed on plant residues and exudates help to stabilize soil carbon, while simultaneously recycling nutrients necessary for plant productivity. Many factors are known to influence community assembly of microbial communities around the host. These include environment, plant species, genotype or health conditions (Fitzpatrick et al., 2018; Xiong et al., 2020; Wagner et al., 2016), microbial interactions, mutualism, or competition (Agler et al., 2016; Hassani et al., 2018), as well as ‘neutral’ processes, such as dispersal limitation, speciation and ecological drift (Rosindell et al., 2011). All these factors are likely to play a role in the establishment of microbiomes and quantitative models including neutral theory, which are becoming more popular for assessing the role of adaptation to different environments and natural selection (Burns et al., 2016; Venkataraman et al., 2015).

Soil chemistry and biology are known to change with depth, yet most studies on belowground plant microbiomes are focused on the top 10 cm of soils since this is where the density of fine roots is often highest (Zhang et al., 2017). Nonetheless, roots of perennial plants may extend meters down into the soil profile where they are important to soil carbon and mineral turnover (York et al., 2022). Therefore, knowledge concerning bioenergy crops and their microbial communities, interactions and functions in deeper soils is needed (de Vries et al., 2017).

Bioenergy crops are being researched as a sustainable alternative to fossil fuels for supplying society's energy needs. To be sustainable, bioenergy cropping systems must maintain neutral or negative CO_2_ emissions (Field et al., 2018), increase ecosystem macro‐ (Fletcher et al., 2011) and micro‐diversity (da C. Jesus et al., 2010), require low or no inputs in terms of fertilizers (Tilman et al., 2006), limit soil erosion and disturbance and be productive on lands that are unsuitable for agricultural food productions (Gelfand et al., 2013; Howe et al., 2021). Research is aimed at understanding how soils and their biodiversity help plants to maintain productive and sustainable biofuel crops with low inputs on lands that are otherwise not well suited for agricultural production.

Here, we present results on fungal and bacterial microbiomes in soils and roots across a 1 m soil‐depth gradient across three bioenergy cropping systems. This research leverages the Great Lakes Bioenergy Research Center's Biofuel Cropping System Experiment (BCSE) at Michigan State's Kellogg Biological Station. Specifically, we aimed to (i) investigate the effect of depth on soil and root fungal and prokaryotic microbiome diversity and structure of poplar, restored prairie and switchgrass, (ii) identify a core set of taxa for each crop and depth and (iii) identify the relationships between microbial taxa, and microbial taxa and the plant host, across the vertical soil niche. We hypothesized that soil microbial diversity would be greatest in surface soils where aboveground organic inputs are concentrated, and would decrease with depth. Given that roots are an important source of carbon belowground, we also hypothesized that microbial community similarity would increase with depth across all three biofuel crops, and overall would be over‐represented by root‐associated taxa—particularly in deep soils. Our results expand knowledge on the fundamental rules that govern microbial communities in bioenergy cropping systems and the significant impact of host plants on soil microbiomes in deep soils.

## EXPERIMENTAL PROCEDURES

### Sampling and metadata collection

In spring 2018, soil cores to 1 m depth (7.6 cm diameter) were taken with a hydraulic probe (Geoprobe 540MT, Geoprobe Systems, USA) at the Kellogg Biological Station (KBS) poplar, switchgrass and prairie research sites. A total of 3 replicate cores were taken at different 5 plots (i.e., block) for each cropping system. Cores were cut by specific depth intervals (0–10, 10–25, 25–50 and 50–100 cm) and for each interval a random of 1 root and soil sample was collected throughout the entire core section. Fine roots were carefully separated from soil with the use of a sieve and fine‐tipped forceps, changing gloves between processed samples, cleansing off attached soil particles. Roots were then washed with a 0.5% Tween 20 solution, rinsed three times with sterile water, and finally wrapped in sterile paper towels and air dried at room temperature. Prior to DNA extraction, roots were powdered in 2 ml tubes using stainless steel beads on a TissueLyser II (Qiagen, USA).

Overall, 60 root and soil samples were collected for each cropping system for a total of 360 samples. Cores were also analysed for total carbon (C %), total nitrogen (N%), sand (%), silt (%), clay (%), pH, PO_4_^3−^ (ppm), K^+^ (ppm), Ca^2+^ (ppm), Mg^2+^ (ppm), and cation exchange capacity (CEC, meq/100 g soil) at each depth and composited by plot (details available at https://data.sustainability.glbrc.org/protocols/158).

### DNA extraction and amplicon library preparation

Genomic DNA was extracted from approximately 0.40 g of dried soils using the PowerMag® Soil DNA Isolation Kit (Qiagen, USA) following the manufacturer's instructions, and from approximately 1 g of fine (ø ≤ 0.5 mm) roots using a CTAB chloroform extraction protocol (Gardes & Bruns, 1993). DNAs were amplified using DreamTaq Green DNA Polymerase (Thermo Scientific, USA) with the primer sets: ITS1f–ITS4 (Gardes & Bruns, 1993; White et al., 1990) and 515F‐806R for Bacteria and Archaea (Caporaso, Lauber, et al., 2010), following a protocol based upon the use of frameshift primers as reported in (Benucci et al., 2019) and originally modified from (Lundberg et al., 2013). PCR products were observed through gel electrophoresis after staining with ethidium bromide and visualized with UV light. Samples were normalized with the SequalPrep Normalization Plate Kit (ThermoFisher Scientific, USA) and pooled together. The generated amplicon library was concentrated to 20:1 with Amicon Ultra 0.5 ml 50 K filters (EMDmillipore, Germany) and purified from primer dimers with Agencourt AMPure XP magnetic beads (Beckman Coulter, USA). We sequenced the amplicon library on an Illumina MiSeq instrument with the v3 600 cycles kit (Illumina, USA).

### Bioinformatic data analysis

Raw internal transcribed spacer (ITS) and 16S reads were evaluated for quality with FastQC (Andrews, 2010). 16S reads were merged with PEAR (Zhang et al., 2014). Forward ITS were used for all downstream analyses. Reads were demultiplexed by barcode sequences in QIIME (Caporaso, Kuczynski, et al., 2010), and Illumina adapters and sequencing primers were removed. Reads were then quality filtered, and trimmed to equal length with Cutadapt (Edgar, 2016; Edgar & Flyvbjerg, 2015; Martin, 2011). After sequence read de‐replication, singletons were removed and sequences clustered into operational taxonomic units (OTUs) based on 97% similarity using the UPARSE (Edgar, 2013) algorithms. Taxonomy assignments were performed in CONSTAX2 (Gdanetz et al., 2017; Liber et al., 2021) against the UNITE eukaryote database, version 8.2 of 4 February 2020 (Abarenkov et al., 2020) and SILVA, version 138 (Quast et al., 2013), respectively. The *‐‐high_level_db* flag in CONSTAX2 was used to identify non‐target taxa as well as OTUs unidentified at the Kingdom level (Bowsher et al., 2020). Non‐target taxa, OTUs not assigned to a Kingdom, and OTUs identified as either chloroplast or mitochondria in either dataset were removed from subsequent analysis.

### Statistical analyses

We first imported summary files from ITS and 16S datasets into the R statistical environment (R Core Team, 2022) and merged them into *phyloseq* objects (McMurdie & Holmes, 2014). We then removed OTUs with less than 10 total sequences (Lindahl et al., 2013; Oliver et al., 2015) to protect against spurious errors, for example, tag switching and artefacts (Carlsen et al., 2012). Before starting the analysis, we explored the library read distribution across samples and according to different variables (Figure S1A,B). We then removed PCR and sequencing contaminants with *decontam* (Davis et al., 2018) using sequence data generated in MiSeq library negative control samples (Figure S1C).

Rarefaction curves for ITS and 16S datasets were generated to visualize variation in sample sequencing depth (Figure S2). The sequence depth was lower for deeper soils than surface soils. To address this, we removed approximately 3% of the samples having fewer library sequences, and we normalized the remaining samples adopting the cumulative sum scaling technique implemented in the *metagenomeSeq* R package (Paulson et al., 2013).

OTU richness (Simpson, 1949) and Shannon's diversity index (Hill, 1973) were calculated with the function ‘specnumber’ and ‘diversity’ in *vegan* (Website). Shannon's index was then rescaled into a 0–1 scale to help comparison across groups using the formula $\mathrm{EH}=1-\frac{H}{\log\left(k\right)}$, with *k* denoting the number of species (i.e., OTUs) and *p*_*i*_ the proportional abundance of species *i*. To test whether depth (i.e., 0–10, 10–25, 25–50 and 50–100 cm) and niche (i.e., root, soil) affected richness and Shannon index we used factorial analysis of variance (ANOVA) (~niche * depth) or Kruskal–Wallis tests when datasets did not meet normality and/or homoscedasticity prerequisites.

Beta‐diversity multivariate analyses were inspired by Anderson and Willis (Anderson & Willis, 2003). In particular, we used: (i) a principal coordinate analysis (PCoA) unconstrained ordination (Kruskal, 1964) followed by a permutational multivariate analysis of variance (i.e., PERMANOVA), to explore similarities between roots and soil samples. (ii) A canonical analysis of principal coordinates (CAP) (Anderson & Willis, 2003) constrained ordination to display differences in community structure explained by the factors in our model and validated with permutation tests to assess the significance of the constraints, ‘cmdscale’ in *vegan* R package (Website). We also calculated adjusted *R* ^2^ as an unbiased measure of the explained variance. We fit environmental vectors onto the CAP ordination with the function ‘envfit’ in *vegan*. (iii) An analysis of multivariate dispersion (Anderson et al., 2006) to test for variance homogeneity among samples and across sample groups. (iv) A taxon‐group association analysis to assess the degree of preference and significance of each OTU for a target group in relation to other groups using function ‘multipatt’ in the *indicspecies* R package (De Cáceres et al., 2010) with the *IndVal.g* methods that incorporates a correction for unequal group sizes. This analysis calculates two species traits: exclusivity (exclusively present in a habitat) and fidelity (present in all samples of that habitat) and an indicator value is calculated based on these traits to assess the extent to which an OTU is an indicator of a treatment or a sample group.

We extracted core OTUs (i.e., frequent, more persistent taxa) across depth for each crop and across crops for each depth following the methodology proposed by Shade and Stopnisek (2019). This approach aids in the identification of core OTUs that differ between crops or depth (all taxa that are core in a group were kept even if not present in other groups). Briefly, abundance‐occupancy distributions were built for each crop and depth and core taxa identified as the set of OTUs that maximize the beta‐diversity resolution (Bray–Curtis similarity) compared to the whole dataset. To inform about stochastically or deterministically recruited community members we then fit neutral models into our OTU distributions to inform about community assembly recruitment processes (Shade & Stopnisek, 2019; Sloan et al., 2006). According to the Neutral Theory, species are ‘neutral’ in the niches they live in. Individual organisms are identical in birth, death, dispersal and speciation rates, and they are only lost or acquired randomly from the source meta‐community. Fitting microbial community composition into a neutral statistical model, which assumes community assembly is driven only by stochastic dispersal and drift, will allow us to delineate the importance of selection and neutral processes and provide a broad insight into mechanisms generating and maintaining community composition (Burns et al., 2016; Venkataraman et al., 2015). Two main coefficients were evaluated in the models: (i) the coefficient of determination (*r* ^*2*^) and represent a measure of the goodness of fit. It ranges from 0 (no fit) to 1 (perfect fit) and is key to assess how important neutral processes are in community structure. (ii) The estimated migration rate (*m*) or the probability that a random loss of an individual in a community is replaced by dispersal from the meta‐community, as opposed to reproduction within the local community, and therefore can be considered a measure of dispersal limitation. The lower the value of *m*, the greater the dispersal limitation impacts community assembly.

To explore co‐occurrence patterns of fungal and prokaryotic OTUs for each crop and depth, we built microbial networks of previously selected core OTUs with the ‘spiec.easi’ function in the *SpiecEasi* R package (Kurtz et al., 2015). To obtain a more accurate network modelling and for known statistical and computational reasons (i.e., rare taxa occurrences can create spurious correlations) (Barberán et al., 2012; Farrer et al., 2019) we built our network on just the core community members obtained as described above. We identified network hubs (OTUs that are central, densely connected with other OTUs in the network) and module hubs (OTUs more densely connected with module's OTUs rather than other OTUs in the network) based on the ratio between within‐module (*Zi*) and between‐module connectivity (*Pi*) and as previously shown (Andrews, 2010; Olesen et al., 2007). We used heatmaps to visualize the connection between proportions of positive and negative intra‐ and inter‐kingdom links (i.e., connections between OTUs), and relative abundances in root‐to‐root connected OTUs, for each crop and depth level.

All analyses and figures were generated in R (R Core Team, 2022) while minimal graphical adjustments to improve figures' visibility were performed in *Inkscape* (Inkscape Project, 2020).

## RESULTS

### Sequencing results

After demultiplexing, we obtained a total of 14,923,238 forward and 8,204,925 reverse sequence reads for ITS, and 21,640,158 forward and 19,917,130 reverse sequence reads for 16S with Phred quality >19, respectively. On average, we generated 38,264 ± 20,136 forward and 21,038 ± 16,500 reverse sequence reads per sample for ITS and 55,917 ± 33,371 forward and 51,465 ± 30,657 reverse 16S sequence reads, respectively. After removing non‐fungal OTUs and contaminants, including filtering out OTUs in positive and negative control samples we were left with 5,123,276 ITS (2794 OTUs) and 17,373,582 16 S (13,855 OTUs) clean sequence reads.

### Microbial alpha diversity

In the ITS dataset, Ascomycota were the most abundant phylum (72.9%), followed by Basidiomycota (10.0%) and the subphyla Mortierellomycotina (1.7%) and Glomeromycotina (1.7%), while in the 16S dataset, the most abundant class was Actinobacteria (28.9%), followed by Alphaproteobacteria (12.3%), Betaproteobacteria (5.7%) and Acidobacteria_Gp16 (5.4%). Archaea in the Thaumarchaeota (1.1%) and Crenarchaeota (<0.1%) phyla were also present but low in abundance.

**FIGURE 1:**
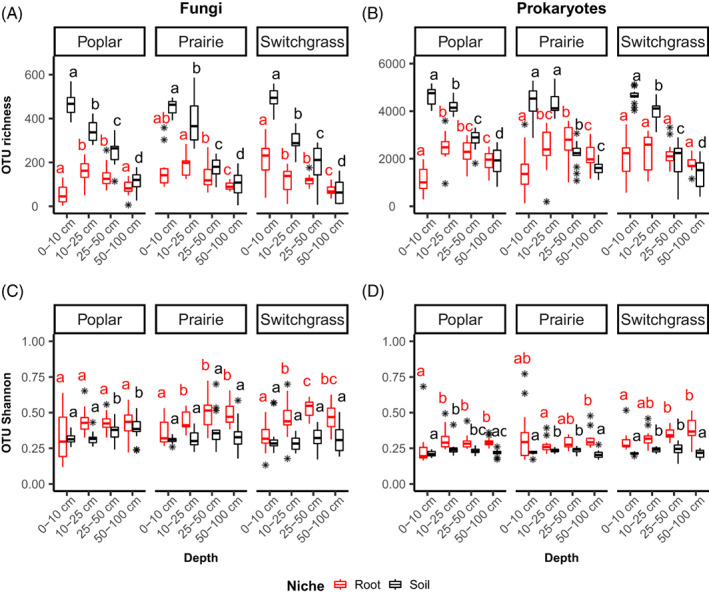
Microbiome alpha diversity metrics and their variations with increasing soil depth. (A) fungi OTU richness; (B) fungi OTU Shannon index; (C) prokaryotic OTU richness; (D) prokaryotic OTU Shannon index. Significant differences within niches (i.e., soil or root) for each crop, across all depths, were tested using Kruskal–Wallis tests, and significant letters obtained using pairwise Wilcoxon tests with *p*‐values corrected using the Benjamini–Hochberg (BH) method. Black stars represent box plot outlier samples.

We found that soil fungal and prokaryotic OTU richness strongly decreases with increasing soil depth in all crops while root communities were less impacted (Figure 1). The Shannon index increased or stayed the same for all crops. Factorial ANOVA (Table S1) showed that niche, depth and their interaction were the main factors driving alpha diversity metrics across crops, and demonstrated that depth impacts microbial richness more strongly than Shannon diversity. In general, communities become less diverse (especially in the soil) and slightly more even (especially in the roots) with increasing depth for both fungi and prokaryotes.

### Microbial beta‐diversity

**FIGURE 2:**
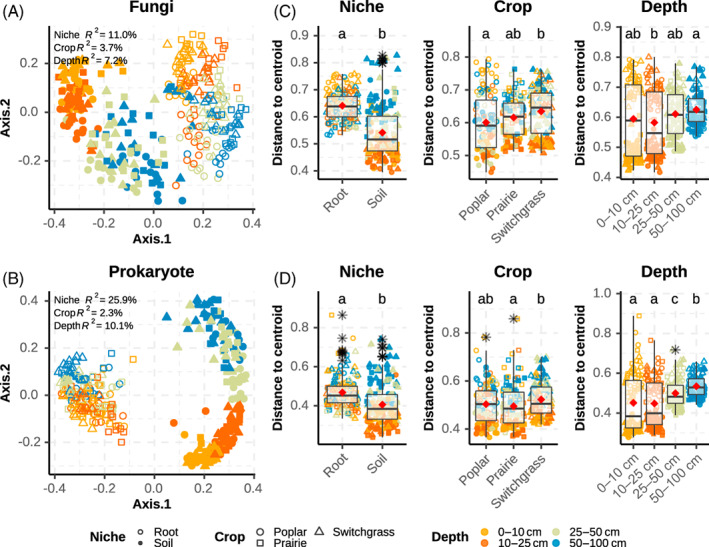
Microbiome community structure shown by principal coordinate analysis (PCoA) and distance to sample group centroid box plots. (A) fungal PCoA; (B) prokaryotic PCoA; (C) dispersion around sample group centroids for fungal communities; (D) dispersion around centroids for prokaryotic communities. Colours represent different levels of depth, shape represents crops, open points represent roots and closed (i.e., filled) points represent soil. In A and B, significant PERMANOVA (“Adonis” permutations [perm.] = 9999, *p* ≤ 0.0001) and related % of variance (*R* ^*2*^ *)* explained by niche, crop and depth sample groups are reported. In C and D, letters represent significant differences between groups (‘permutest’, perm. = 9999, *p* ≤ 0.0001), coloured jittered points represent samples, while black stars represent outlier samples.

Fungal and prokarayotic communities clustered mainly by niche (i.e., soil vs. root), depth and ultimately crop, as displayed in the PCoA ordination graph (Figure 2). The same trends were detected by PERMANOVA (i.e., ‘adonis’, permutations [perm.] 9999), which showed significant differences (*p* ≤ 0.0001) in community structure between roots and soil samples (i.e., niche factor) accounting about 11% and 26% of the variation for fungi (Figure 2A) and prokaryotes, respectively (Figure 2B). Depth was the second significant factor in terms of explaining variation affecting microbial communities (7% fungi and 10% prokaryotes) followed by crop (about 4% fungi and 2% prokaryotes).

In addition, we detected significant dispersion (*p* ≤ 0.0001) around centroids (i.e., ‘betadisper’ and ‘permutest’, perm. 9999) in niche, crop, and depth samples for fungal (Figure 2C) and prokaryotic (Figure 2D) communities. Fungal and prokaryotic root samples showed significantly higher average dispersion than soil samples (i.e., higher heterogeneity between samples), but soils showed a wider distribution implying there is greater variation between centroids in soil samples compared to roots. Interestingly, a significant dispersion effect was present between samples at different crops and depths, with deeper soils having a higher dispersion and narrower distribution.

**FIGURE 3:**
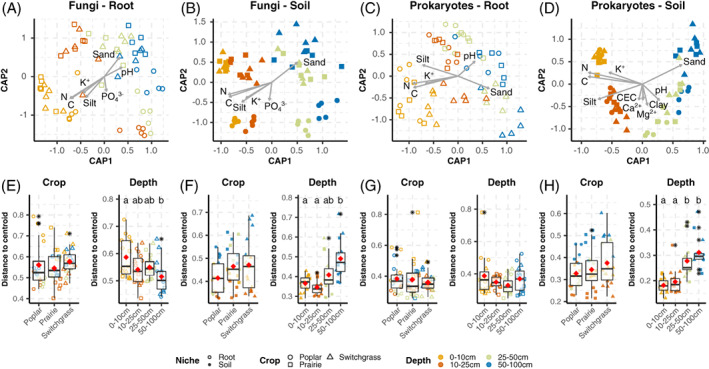
Microbiome community structure shown by constrained analysis of principal coordinates (CAP) and distance to sample group centroid box plots. A, root fungi; B, soil fungi; C, root prokaryotes; D, soil prokaryotes. Root samples are visualized as open, while soil samples as solid points. Circles represent poplar, triangle represents switchgrass, and square represents prairie samples. Colours represent different levels of depth, shape represents crops, open points represent roots, and closed (i.e., filled) points represent soil. Significant environmental vector (‘envfit’, permutations [perm.] = 9999, *p* ≤ 0.05) were plotted in the ordination graph (see Experimental Procedures section for units). Box plots of sample dispersion around centroids for crop and depth centroids (E–H) are also shown underneath each ordination graph. Letters represent significant differences (*p* ≤ 0.0001) in average dispersion from centroids (‘permutest,’ perm. = 9999). Stars represent outlier samples.

For an in‐depth understanding of the effects that crop species and soil depth had on the microbial communities, we analysed root and soil separately with canonical analysis of principal coordinates (CAP) (Figure 3) fitted to environmental vectors. In this case, samples clustered mainly by depth (i.e., CAP1) both in fungal (Figure 3A,B) and prokaryotic (Figure 3C,D) communities, but tighter clusters were visible in the soil compared to the root communities. A separation by crops (i.e., CAP2) is also detectable in the CAP ordination, and more visible for fungi where poplar samples lie further apart than the other crops, compared to the prokaryotes. Indeed, depth showed the greatest significant effect (*p* ≤ 0.0001) for both fungal and prokaryotic communities, followed by crop and the interaction between the two (Table 1). In particular, the variance explained (i.e., adjusted *R* ^*2*^) by depth was higher in the soil (about 24% for fungi and 50% for prokaryotes) compared to the roots (about 13% for fungi and 16% for prokaryotes). The interaction factor (i.e., crop: depth) explained a low amount of variance in all datasets, ranging from about 3% of the soil prokaryotes to 5% of root fungi (Table 1). We found non‐significant dispersion around centroids (variances) between crops in all communities, as shown in the box plots of Figure 3E–H, representing the distance to centroids distribution for each sample. However, we found significant dispersion (*p* ≤ 0.0001) around centroids between samples of different depths for root fungi, soil fungi and soil prokaryotes, whereas dispersion was not significant for crops and depth for soil prokaryotes (Figure 3G). Sample dispersion decreased with increasing depth in the roots (i.e., root communities became more similar to each other with increased depth), but stayed constant or increased in soils (i.e., communities were more different from each other in deeper soils). Fitted environmental vectors showed that soil microbial communities towards the surface correlated with higher total carbon (C%), nitrogen (N%), phosphorus (PO_4_^3−^), potassium (K^+^) and silt, whereas communities of deeper soils correlated with increased pH and sand content. Interestingly, PO_4_^3−^ fit significantly into fungal ordinations, while calcium (Ca^2+^) and magnesium (Mg^2+^) only fit into the prokaryotic ordinations, with higher levels towards the soil surface.

**TABLE 1:** Constrained analysis of principal coordinates (CAP) of soil and roots, fungal and prokaryotic, communities

Group	Factor	CAP	
		*F*/*t*	Adjusted *R* ^2^
Fungi–roots	*Crop*	4.0162_(2,57)_***	0.0931
	*Depth*	3.8513_(3,56)_***	0.1271
	*Depth:Crop*	1.6413_(6,48)_***	0.0517
Fungi–soil	*Crop*	3.3111_(2,57)_***	0.0728
	*Depth*	7.1076_(3,56)_***	0.2375
Prokaryotes–roots	*Crop*	4.0065_(2,57)_***	0.0930
	*Depth*	4.6826_(3,56)_***	0.1585
	*Depth:Crop*	1.4213_(6,48)_**	0.0337
Prokaryotes–soil	*Crop*	1.2017_(2,57)_	0.0068
	*Depth*	20.358_(3,56)_***	0.4961
	*Depth:Crop*	1.6466_(6,48)_***	0.0321

*Note*: Partial model effect sizes (*F*‐ratio) and *t‐*statistic (*t*) were based on permutational ANOVA with 9999 permutations. Adjusted *R* ^*2*^ values represent a measure of explained variance (%). Only significant partial models from the full model *crop + depth + crop:Depth* are shown. Signif. Codes: “0.001” (***), “0.01” (**), “” (not significant).

Chemistry data alone indicate that soil N and C%, as well as the amount of K^+^, (*p* ≤ 0.05) decreased significantly with increasing depth. Soil micronutrients (i.e., Ca^2+^ and Mg) accumulate at median soil depths. Soil texture changed with depth, with % sand increasing considerably in deeper soils. An inverse pattern was seen for silt, and was statistically significant (*p* ≤ 0.05) in prairie and switchgrass but not in poplar. In addition, strong positive correlations were found between Ca^2+^ and Mg^2+^ contents and cation exchange capacity (CEC) values (Figure S3).

**FIGURE 4:**
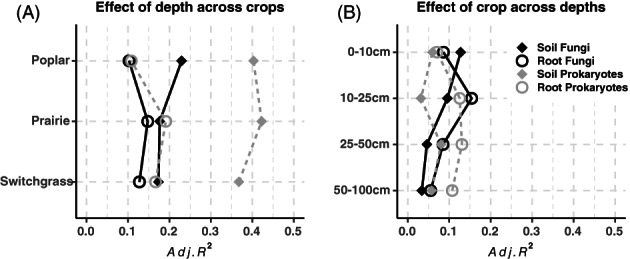
Effect of depth and crop species on root and soil microbiomes. Each point represents the adjusted *R* ^2^ as a measure of the effect of depth (A) across different crops, or the effect of crop (B) at different soil depths. *R* ^2^ values were calculated using CAP (constrained analysis of principal coordinates) on dataset divided by crop (A), for each niche and organism group (i.e., soil fungi, root fungi, soil prokaryotes, root prokaryotes), with the model: *Capscale(otu ~ depth*, *dist = “bray”)*, and the dataset divided by depth (B) for each niche, with the model: *Capscale(otu ~ crop*, *dist = “bray”)*. All CAP models were tested for significance using permutational ANOVA (“permutest”, permutations = 9999, *p* ≤ 0.05) before inclusion in the plot.

The adjusted *R* ^*2*^ form CAP analysis performed on individual groups of samples (Figure 4A) clearly showed the effect of depth on community structure was generally higher for soils than roots, particularly for prokaryotes. Depth affected poplar soil fungi the most and root fungi the least. On the other hand, the effect of crop was higher in root than in soil communities and generally higher close to the surface with respect to deeper soils in the fungal communities (Figure 4B). For example, the highest effect of crop was detected for fungal roots communities at 10–25 cm.

### Neutral models, core taxa and microbial networks

**FIGURE 5:**
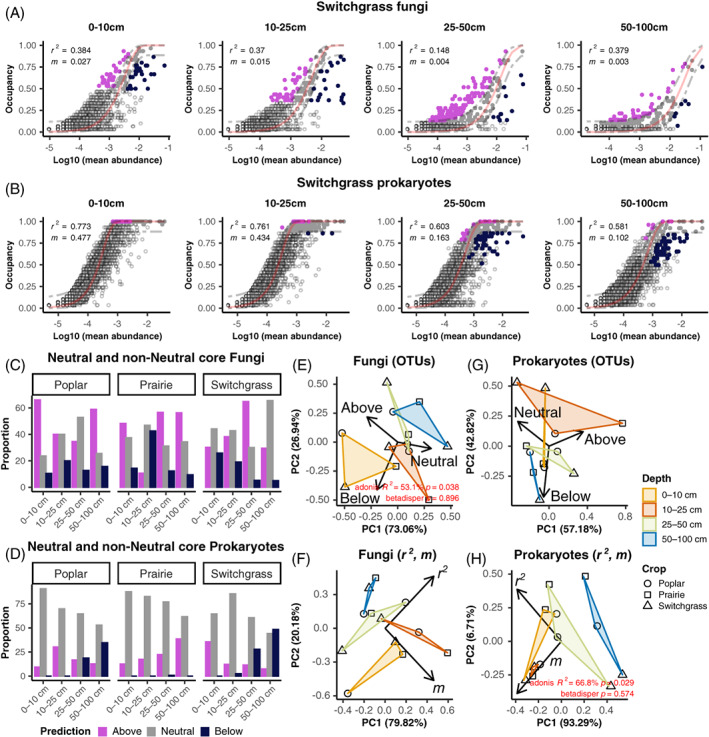
Neutral model fit line on log10 abundance‐occupancy OTU plot for: A, fungal and B, prokaryotic communities of switchgrass at each depth (poplar and prairie shown in Figure S4). Filled points represent the core while empty points are other OTUs. Proportion (%) of neutral, above, and below prediction of fungal (C) and prokaryotic (D) core OTUs, at different depths and for each crop, see colour coded legend for groups. Model fit (*r* ^*2*^) and migration rate (*m*) are also plotted into the graphs for each model. Principal coordinate analysis (PCA) of all fungal (E) and prokaryotic (F) OTUs identified as neutral, below or above the prediction by the neutral model fit, at different depths and for each crop. PCA of *r* ^*2*^ and m values for each neutral model obtained at each depth and for each crop for fungal (G) and prokaryotic (H) communities. Core OTUs in the models are highlighted according to their predictions in respect to the model fit (see legend D). Significant differences (*p* ≤ 0.0001) between groups were assessed using PERMANOVA (‘adonis’ *R* ^*2*^, permutations [perm.] = 9999) while homogeneity of group variances using *betadisper* and permutational ANOVA (‘permutest’, perm. = 9999).

Neutral processes could help drive community assembly and maintenance. To assess the importance of neutral and non‐neutral processes, for example, microbial interactions or dispersal, we fit our data into a neutral assembly model (Figure 5A,B, Figure S4). We found the proportion of neutral, above, and below model prediction OTUs were similar across depths and in the different crops (Figure S5). However, when just the core fungal and prokaryotic OTUs (defined here as the minimum OTU set that preserve the same community structure) (Shade and Stopnisek, 2019) were selected separately, some interesting trends were found. In the fungal communities, neutral OTUs (i.e., OTUs driven by drift) were more abundant in deeper soils (Figure 5C, D, E, G). In surface soils, OTUs above (i.e., OTUs selected or maintained by the host) or below (i.e., these are OTUs selected against by the host, or dispersal limited) the model predictions were more abundant (Figure 5A, C, E), particularly in poplar and switchgrass. To detect if the proportions of core OTUs classified by the neutral models were grouped according to crop or depth, we performed a PCA and significant differences between groups tested with PERMANOVA. The proportion of neutral, above and below prediction fungal OTUs statistically significantly separate by depth, which explained about 53% of variation in data. In the prokaryotic communities, we can clearly see a higher number of OTUs below the model prediction in deeper soils and a lower number of neutral OTUs (Figure 5B, D, G), especially in poplar and switchgrass.

In general, microbial patterns in prairie systems were less distinguished, perhaps due to the diverse nature of prairies in terms of plant species present and their associated microbiomes. Regarding the neutral models goodness of fit (*r* ^*2*^), the models based on the prokaryotic communities showed on average a higher fit compared to the fungal ones (Figure S4) implying a higher importance of neutral processes in structuring these communities. Neutral fit was also generally lower in deeper soil samples compared to the surface in both communities. In addition, the migration rate (*m*) was on average higher in soil samples closer to the surface and lower in deeper soils, for both fungi and prokaryotes. Low *m* values suggest higher influence of dispersal limitation in community assembly (Figure 5F, H). Again, we used PCA and PERMANOVA to detect significant differences in *r* ^*2*^ and *m* rate between crops or depths. We found that only in the prokaryotic communities, *r* ^*2*^ and *m* significantly separated by depth, explaining about 66% of variation in the data, indicated that neutral processes have greater consequences for community assembly in deeper samples compared to more shallow ones.

**FIGURE 6:**
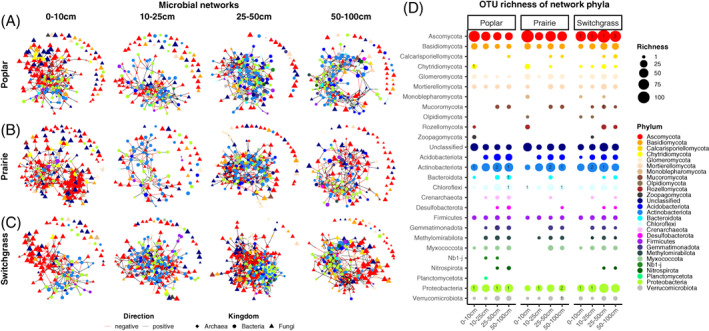
(A) Fungal‐prokaryotic covariance networks of the core taxa identified at different depths (0–10, 10–25, 25–50 and 50–100 cm) for A, poplar; B, prairie: and C, switchgrass. (B) Balloon plot of taxonomic richness of all phyla present in the networks. Bubbles represent the number of operational taxonomic units (OTUs) for each taxonomic group, larger bubbles mean higher richness. Unclassified fungi at phylum level are also included in the plot.

Since the core taxa appear to follow specific trends or relationships across depths (i.e., 0, 25, 50 and 100 cm) and to particular plant hosts, we used these taxa to explore covariance networks (Figure 6A–C) to identify potential interactions between the members of the communities. Microbial networks showed quantitative and qualitative shifts in diversity across soil depth and crop species. The number of Ascomycota fungal OTUs decreased with depth while bacterial, Actinobacteriota and Proteobacteria increased with depth. This was most pronounced in poplar and prairie systems, but not in switchgrass, where samples at 25 and 50 cm depth were the most diverse (Figure 6D). Bacterial OTUs within the Actinobacteriota, Proteobacteria, Chloroflexi and fungi in Ascomycota and Chytridiomycota were defined as network hubs (Table S2). Interestingly, only a single fungal hub was present in poplar, and a few in switchgrass—which was comprised exclusively by bacteria, as reported by the number within the bubbles in Figure 6D.

**FIGURE 7:**
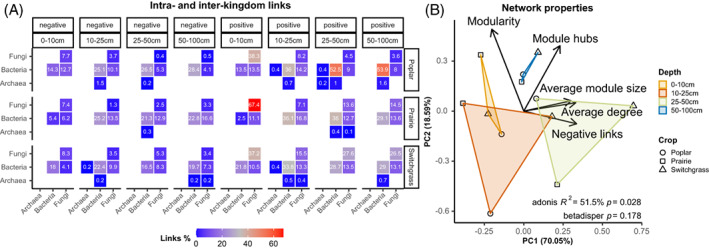
(A) Heatmap showing the proportions of positive and negative intra‐ and inter‐kingdom links (i.e., connections between operational taxonomic units [OTUs]) for each crop and depth level. (B) Principal component analysis (PCA) of the most important network properties identified using random forest. Significant differences between groups (*p* ≤ 0.05) were assessed using PERMANOVA (‘Adonis’ *R* ^*2*^, permutations [perm.] = 9999) while homogeneity of group variances using *betadisper* and permutational ANOVA (‘permutest’, perm. = 9999, *p* ≤ 0.05).

Positive and negative intra‐ and inter‐kingdom links showed that fungi–fungi links decreased with increasing depth in all crops, while bacteria–bacteria links increased but stayed more or less the same in switchgrass (Figure 7A). Fungi–bacteria links decreased with depth in poplar but not in prairie and switchgrass. Regarding network complexity, several network properties increased with depth until 50 cm, and then decreased (Table S3).

When we look at the abundance of positive and negative intra‐ and inter‐kingdom root‐to‐root links (i.e., the higher the abundance the more the links are between root OTUs), we discovered that root‐to‐root and fungi–fungi links decrease with increased depth in all crops (in deeper soil there are more soil‐to‐root links compared to the surface), except for switchgrass were differential patterns were not very clear (Figure S7). Root‐to‐root bacteria–bacteria links increase with increased depth (in deeper soil there are more root‐to‐root links compared to the surface). Positive and negative root‐to‐root fungi–bacteria links decrease in Poplar, while seems to increase or not having a defined trend in prairie and switchgrass (Figure S7). Interestingly, the highest positive fungi–fungi root‐to‐root abundance was detected for prairie at 0–10 cm, while the highest bacteria–bacteria abundance for poplar at 50–100 cm. At phylum level, there was an increase of root‐to‐root links between OTUs within Proteobacteria and Actinobacteriota, and a decrease within Ascomycota, for all crops (Figures S8, S9).

Five network properties were able to statistically discriminate (*p* ≤ 0.05) between the networks across depth but not across crops (Figure 7B). Modularity and the number of module hubs were higher in deeper soils. Average module size and average degree were correlated one another and together with negative links higher in soils at 25–50 cm depth (Figure 7B).

## DISCUSSION

In this study, we assessed the major forces that regulate the dynamics of soil microbial communities in plant–soil environments along a vertical niche belowground. Leveraging long‐term field‐scale replicated experiments, we were able to analyse several aspects of these plant‐associated microbiomes along a 1‐m soil‐depth gradient for poplar, prairie, and switchgrass biofuel crops in replicated plots. We demonstrate a significant vertical niche in soil and root compartments, and consider the drivers and consequences of this vertical diversity gradient in roots and bulk soils.

### Differences between root and soil microbiomes

As documented in other studies, we report that microbial communities in roots are less diverse and quite distinct from those in bulk soil (Goldmann et al., 2016; López‐Angulo et al., 2020). We also found microbial communities are variably distributed at a fine scale. Yet, alpha diversity in roots and soils follow different trends along the sampled depth gradient. Soil carbon, together with pH and nitrogen, appear to be the most important factors explaining microbial biomass and functional diversity in soil ecosystems (Fierer & Jackson, 2006; Fierer, 2017; Bastida et al., 2021). As previously suggested (Celestina et al., 2019; Mundra et al., 2021; Yokota et al., 2022), greater carbon stocks and nutrient content of surface soils may account for significantly greater microbial diversity in surface soils, as we found across all bioenergy crops. Aboveground litter contributes diverse organic matter to mineral soils, but these inputs decrease significantly with increasing soil depth where carbon from roots becomes increasingly important in driving heterotrophic soil food webs. Greater nutrient, oxygen and water availability, as well as higher microclimatic variation may also contribute to more ecological niches in surface soils compared to deeper soil, thus, enabling the support of greater microbial diversity (Mundra et al., 2021).

### The belowground vertical niche

While drastic differentiation within bacterial and fungal communities are known to exist between organic and mineral soil horizons (Peršoh et al., 2018), our study focused on soil below the organic horizon and also found significant differentiation. Previously, ectomycorrhizal fungi were shown to differentiate along a vertical niche (Dickie et al., 2002). Although poplar was the only ectomycorrhizal host sampled here, we expected that other microbial guilds would follow similar patterns of differentiation, and this is what we found. Decreasing microbial species richness with increasing soil depth is well documented in soil microbial ecology studies across different ecosystems (Zhang et al., 2017; Jiao et al., 2018; Hao et al., 2020; Frey et al., 2021). It has been also shown that variable gradients of carbon, nitrogen, pH and oxygen usually correlate with declines in microbial biomass and diversity (Fierer et al., 2003; Schlatter et al., 2018; Ren et al., 2022). For instance, the abundance and diversity of bacterial communities in a permafrost zone were both found to decrease to a 70‐cm depth, and abiotic factors, such as soil temperature, carbon, nitrogen, phosphorus, moisture and clay content, respectively, were the most significant factors driving bacterial community diversity (Ren et al., 2022). Yet, these factors often co‐vary with depth, making it challenging to disentangle the main drivers without more controlled studies.

### Core microbiome

Taxa that are consistent across samples and datasets constitute the core microbiome, and can be defined by specific abundance‐occupancy distributions (Shade and Stopnisek, 2019). Core microbiome members are hypothesized to be functionally significant to their niche. To better understand the ecological and potentially functional relationships shared between soil microbes and plant rhizospheres, we identified core microbiome members across niches and depths. We fit these microbial distributions into a neural model to predict the importance of selection and drift in organizing these communities. Together, our data showed the fungal communities of sampled bioenergy crops in the surface soil layers (e.g., 0–25 cm) have a higher number of core OTUs that are above or below the neutral model predictions, while neutral OTUs are higher in the deep layers (50–100 cm). This is in contrast with what was found by Powell et al. who investigated the role of deterministic and stochastic processes in vertical soil horizons at 183 sites across Scotland, and measured high stochasticity in fungal communities on the surface soils (Powell et al., 2015). However, Powell et al. analysed natural sites to a depth of 75 cm, rather than agricultural fields, which may explain the differences in the results.

In our analysis, most of the fungi on the soil surface undergo selective processes, mediated by the host, or by the microbes themselves, and finally occupy and maintain a specific occupied niche—coexistence through niche differentiation. In contrast, in deeper soils, we find more fungi that follow a model of passive dispersal and ecological drift. This phenomenon causes species abundances to randomly vary, reducing diversity within communities and increasing differences between communities. In harsh environments, such as deeper soils where resources are limited, an equalizing mechanism that reduces differences in relative fitness among species has also been proposed to maintain species coexistence (Kim & Ohr, 2020).

Interestingly, we saw a different pattern in the prokaryotic communities. A higher number of OTUs below the model prediction was observed in the deeper soil layers while the number of neutral OTUs decreased with increasing soil depth. For instance, neutral, above‐, and below‐prediction core OTUs proportions clustered significantly by depth in fungal communities, but not in the prokaryotic communities. Depth was a statistically significant factor that influenced model fit and migration rate with decreased depth for the core prokaryotic communities. We speculate that the unicellular nature of prokaryotic organisms, including traits of motility, and dispersal via soil hydrology, differentiates the macroecology of bacteria from that of filamentous fungi. Indeed, It has been shown that the soil water content correlates with the richness of soil microbial communities (Jonas et al., 2015; Aung et al., 2018) and that motility impacts root colonization by bacteria (Knights et al., 2021). It is also important to consider that moisture content and temperature are generally more stable in deeper soils compared to surface soils.

### Microbial networks

Microbial networks are a way to statistically assess the strength of interactions and linkages between taxa within a dataset. We assessed microbial networks based on identified core microbiomes and found that deeper soils consist of more dense networks that have higher connectivity. A similar approach was recently used in grassland ecosystems (Upton et al., 2020), who found that fungal and bacterial networks of native plants were more connected at lower soil depths, even if there were fewer nodes. Higher connectivity in deeper soil may be due to the relative importance that root C inputs have on microbial activity at deeper rooting depths. In addition, since deeper soil depths harboured less diverse fungal communities, we expected to see larger networks as more OTUs were shared between samples across niches and depths. In all crops, we detected the general trend of decreasing fungal and increasing prokaryotic core OTUs with increasing depth in all crops. Our results correlate with what obtained by Yao et al. used phospholipid fatty acids (PLFA) analysis to investigate factors influencing soil microbial communities in temperate grasslands of northern China (Yao et al., 2018). They also found that fungi were more abundant in the surface while prokaryotes in deeper soils, highlighting another fundamental difference between patterns of fungal and bacterial community diversity.

Results from our study show that fungi–fungi links decreased with increasing depth in all crops while bacteria–bacteria links increased with depth, or remained fairly constant in the case of switchgrass. The diversity of core fungi in the roots decreased with depth, while that of bacteria increased. Microbial network modularity, number of hubs, average module size, average degree and negative links were statistically significantly separated by depth, with more modules (and more module hubs) in deeper soil implying they may have greater resistance to environmental changes compared to communities in upper soil layers. These results contrast with those found by Mundra et al. (2021), where upper mineral soil harboured a higher modularity and also more inter‐kingdom links compared to the above organic layers or deeper mineral layers. Nonetheless, the differential partitioning of core fungal and bacterial networks with soil depth across all three bioenergy species highlights the important contribution of plant communities on deep soil microbial communities, whose functions are critical to the sustainability of these bioenergy cropping systems.

## CONCLUSIONS

Microbial communities are a key component of any agricultural system and their role in biogeochemical cycling is well known. However, the extent that these communities vary in diversity and structure with soil depth, and their relationships with the host, are less studied. In this study, we found that soil depth has a major impact on soil and root microbiomes, with soil microbial diversity correlating with carbon availability and decreasing with soil depth. Communities in the deeper soil were less diverse, but were also less heterogeneous in the roots and more heterogeneous in the soils. In deeper soils, roots appear to be a major factor generating niche breadth for microbial life to persist and function, further impacting soil structure and functioning. Stochastic process described the prokaryotic communities more accurately than they did fungal communities, and there was a significantly different model fit for fungi and bacteria across this vertical soil niche. Overall, neutral fungal core taxa were higher in deeper soils, which were dominated by dispersal‐limited prokaryotes, underlying the biological, ecological and morphological differences present in these Kingdoms. Co‐occurrence networks were more connected and modular in deeper soils, indicating a higher rate of interdependence in more confined oligotrophic soil environments. Taken together, we provided a novel understanding of soil microbiomes and their interactions in connection to different bioenergy hosts and cropping systems. This knowledge is key to leveraging plant microbiomes for the many functions they provide in the environment to support cleaner, and more sustainable agricultural and energy economies.

## AUTHOR CONTRIBUTIONS

**Gian Maria Niccolò Benucci:** Methodology, Illumina library preparation, software, bioinformatics, data analysis, validation, data curation, database, supervision, writing ‐ original draft preparation, writing ‐ reviewing and editing. **Pedro Beschoren da Costa:** Methodology, DNA extraction, data analysis, writing‐ reviewing and editing. **Xinxin Wang:** Methodology, DNA extraction. **Gregory Bonito:** Conceptualization, validation, supervision, project administration, funding acquisition, investigation, writing ‐ original draft preparation, writing ‐ reviewing and editing.

## CONFLICT OF INTEREST

Authors declare no competing interests in relation to the work described.

## Supporting information


**Data S1:** Supporting information

## Data Availability

Raw ITS and 16S sequences*. fastq* reads were deposited to the sequence read archive (Leinonen et al., 2011) under the bioproject no. PRJNA751075. Data sets, and R code developed to analyse them, are accessible at https://github.com/Gian77/Scientific‐Papers‐R‐Code/tree/master/Benucci_etal_2022_DeepSoilMicrobiome.
