# Supplementary material for: Stochastic and deterministic processes shape bioenergy crop microbiomes along a vertical soil niche
Source: Environ Microbiol. 2022 Nov 24;25(2):352–66. doi: 10.1111/1462-2920.16269 (PMC10099798; doi:10.1111/1462-2920.16269)
Supplement: Supplementary file 1 — Data S1: Supporting information [file EMI-25-352-s001.pdf]

Supplementary Materials

Figure S1 Distribution of illumina sample libraries. A) Fungi soil and root communities, B) Prokaryotes soil and root communities, C) TRUE contaminant OTUs (red points) as identified using 0.5 prevalence in the negative controls using the *decontam* R package. Dotted red lines in A and B represent the overall mean for the community. Soil depth ranges, 0-10, 20-25, 25-50, and 50-100 are in cm.

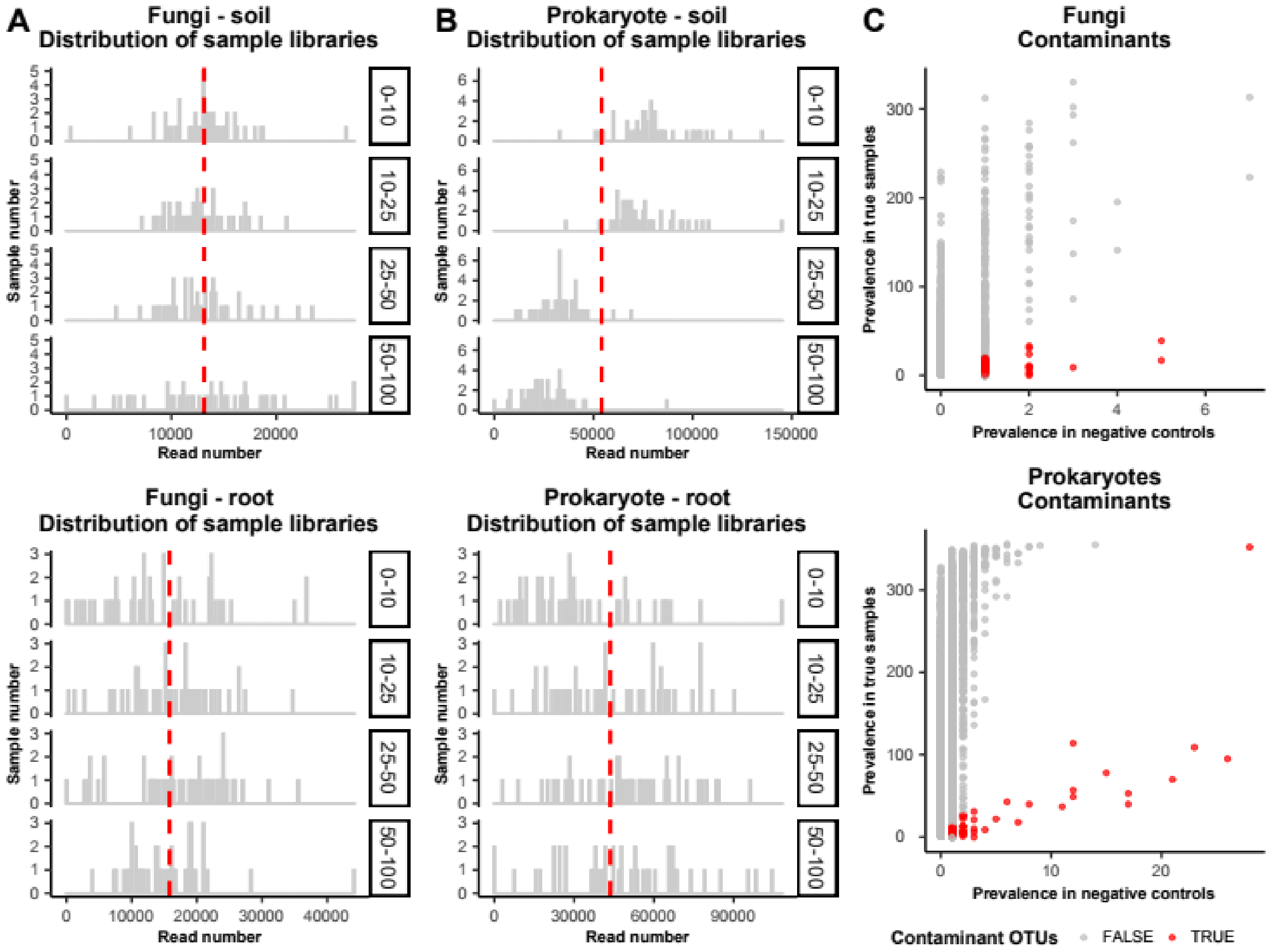

Figure S2 Rarefaction curves for A) fungal and B) prokaryotic communities.

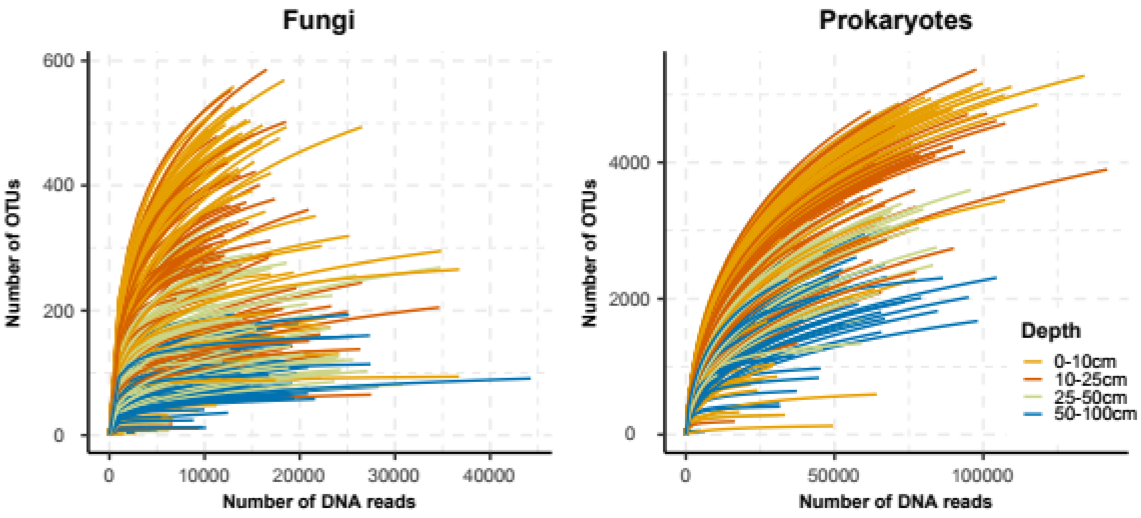

Figure S3 Soil chemistry. A) Soil nutrients variation with increasing depth and B) spearman correlation between elements. Significant differences (P ≤ 0.05) were pairwise Wilcoxon tests with Bonferroni corrected P-values.

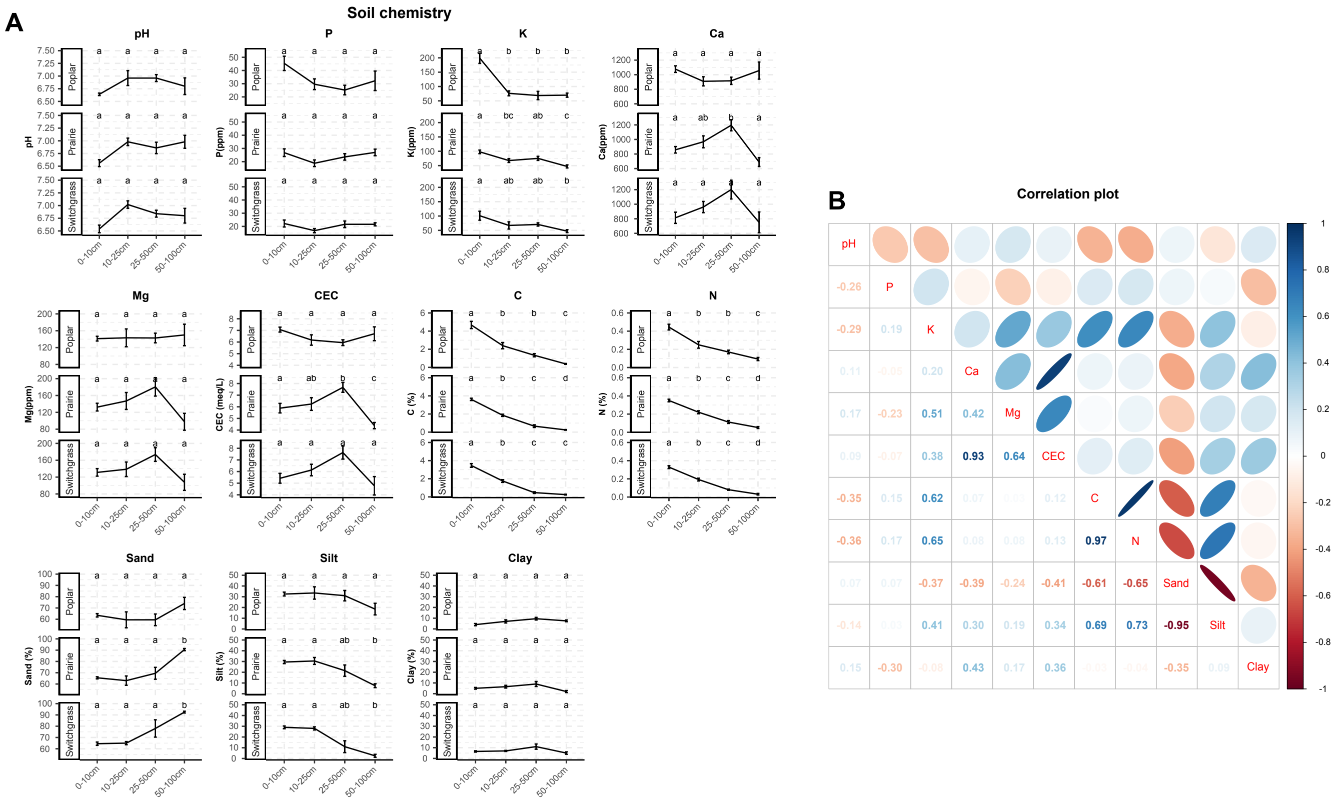

Figure S4 Neutral models fit line on log10 abundance-occupancy OTU plot. Datasets were subsetted by crop and depth levels: A) poplar fungi, B) poplar prokaryotes, C) prairie fungi, D) prairie prokaryotes. Model fit (r^2) and immigration rate (m) are also plotted into the graphs for each model. Filled points represent the core while empty points are other OTUs. Proportion (%) of neutral (grey), above (purple), and below (dark purple) model prediction are shown.

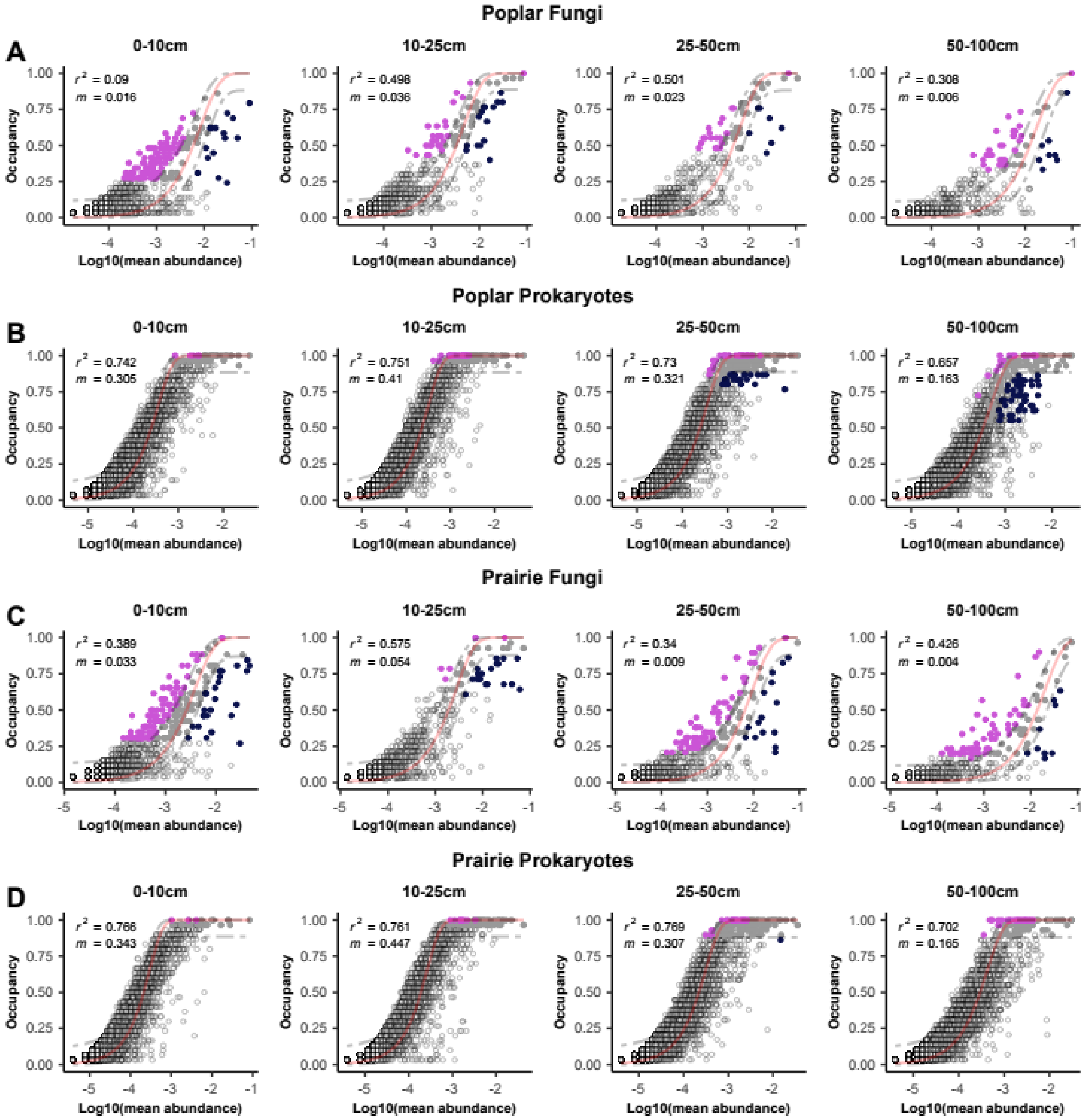

Figure S5 Proportion of all the neutral, above, and below model prediction OTUs for A) fungal and B) prokaryotic communities.

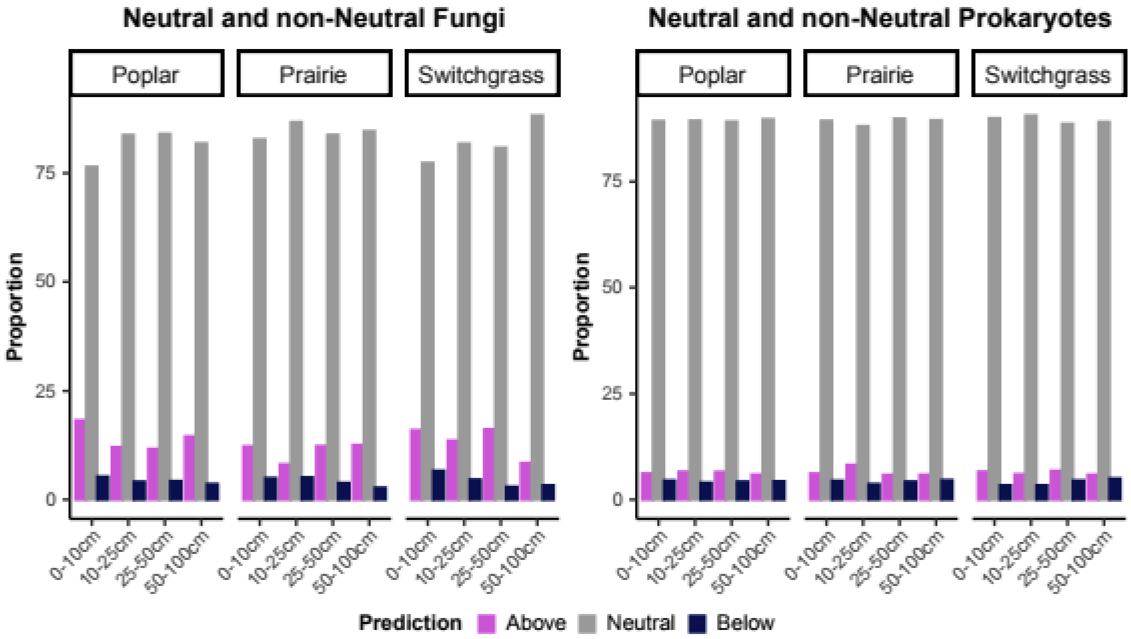

Figure S6 Balloon plot of relative abundance of all phyla present in the networks. Bubbles represent the cumulative relative abundance of all OTUs for each taxonomic group. Relative abundance value are also reported on top of the bubbles (larger bubbles mean higher relative abundance) and sum up to 100% at each depth. For completeness, unclassified fungi at phylum level are also included in the plot.

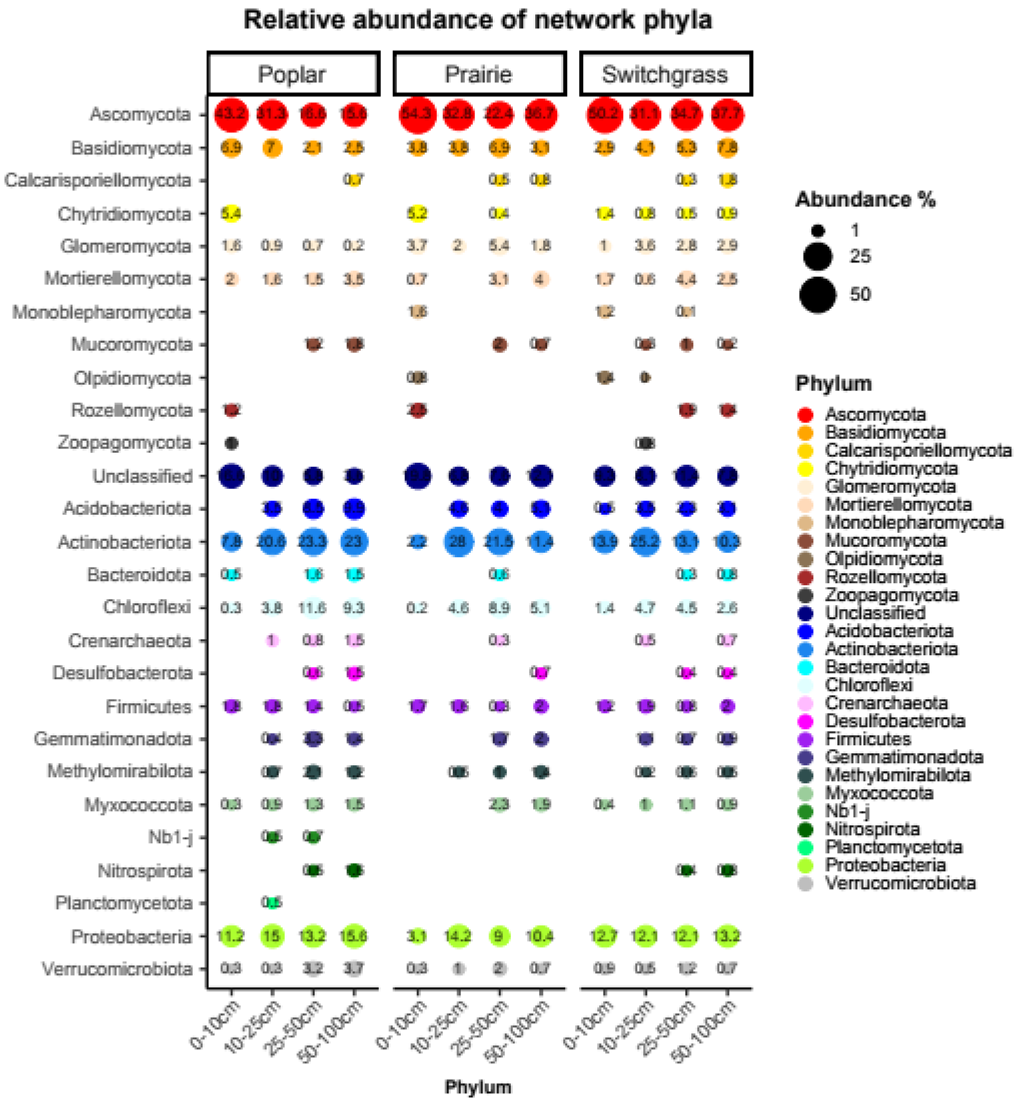

Figure S7 Heatmap showing the abundance of positive and negative intra- and inter-kingdom root-to-root links (see Material and Methods section in main text). The higher the abundance the more the links are between root OTUs, the lower the abundance the more the links are between soil OTUs.

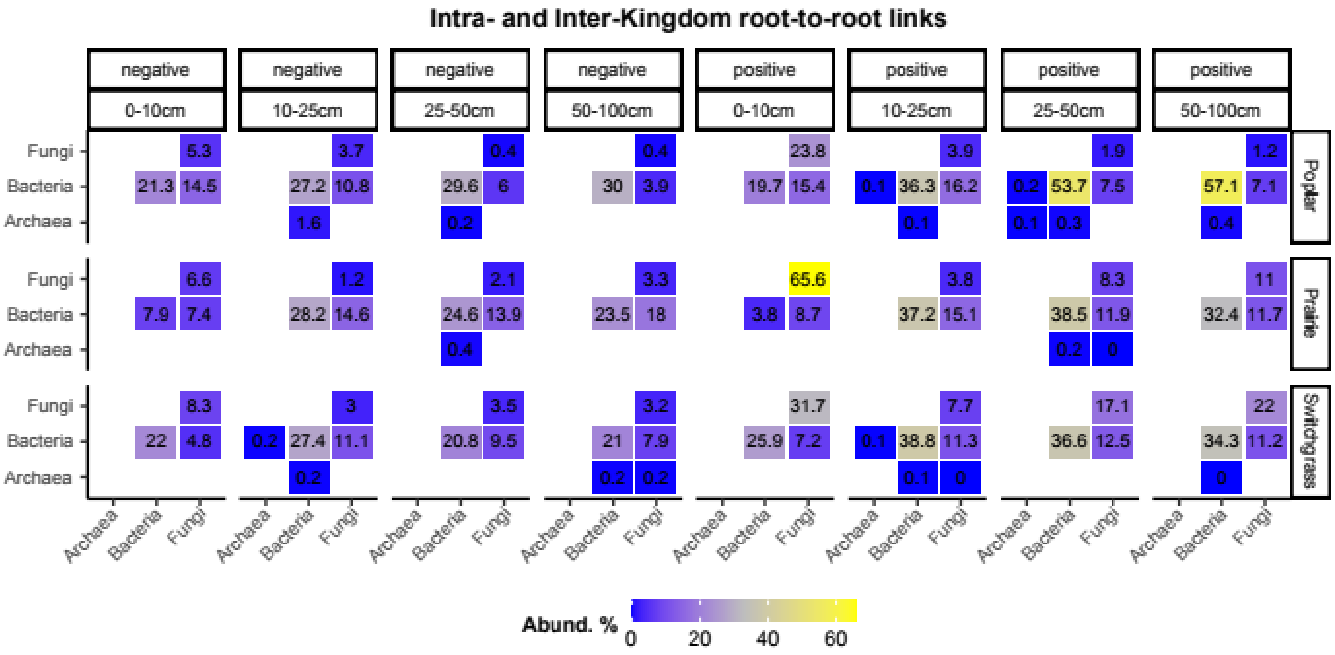

Figure S8 Heatmap showing the proportions of positive and negative intra- and inter-Phylum links (i.e. connections between OTUs) for each crop and depth level.

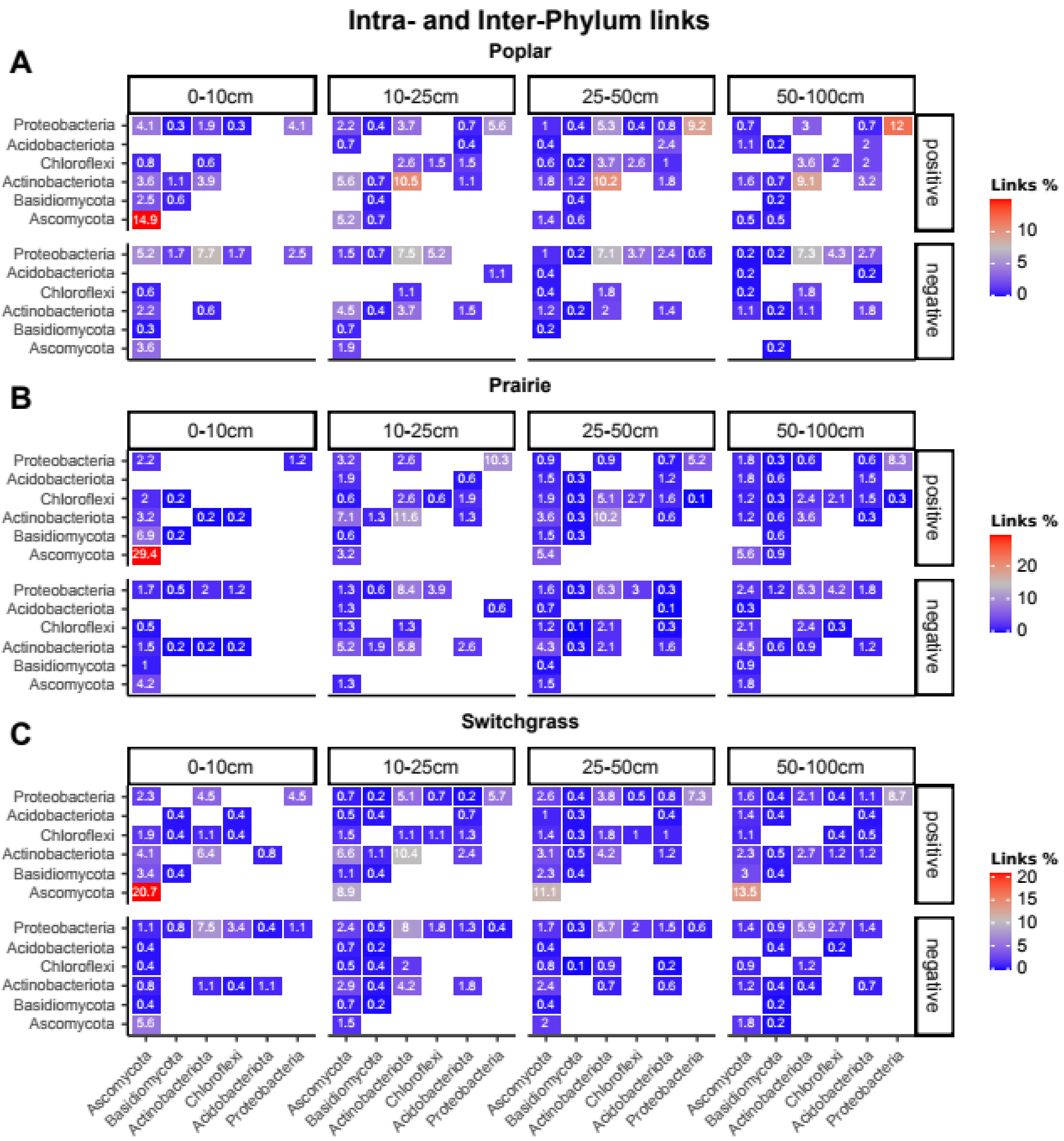

Figure S9 Heatmap showing the abundance of positive and negative intra- and inter-Phylum root-to-root links. The higher the abundance the more the links are between root OTUs, the lower the abundance the more the links are between soil OTUs.

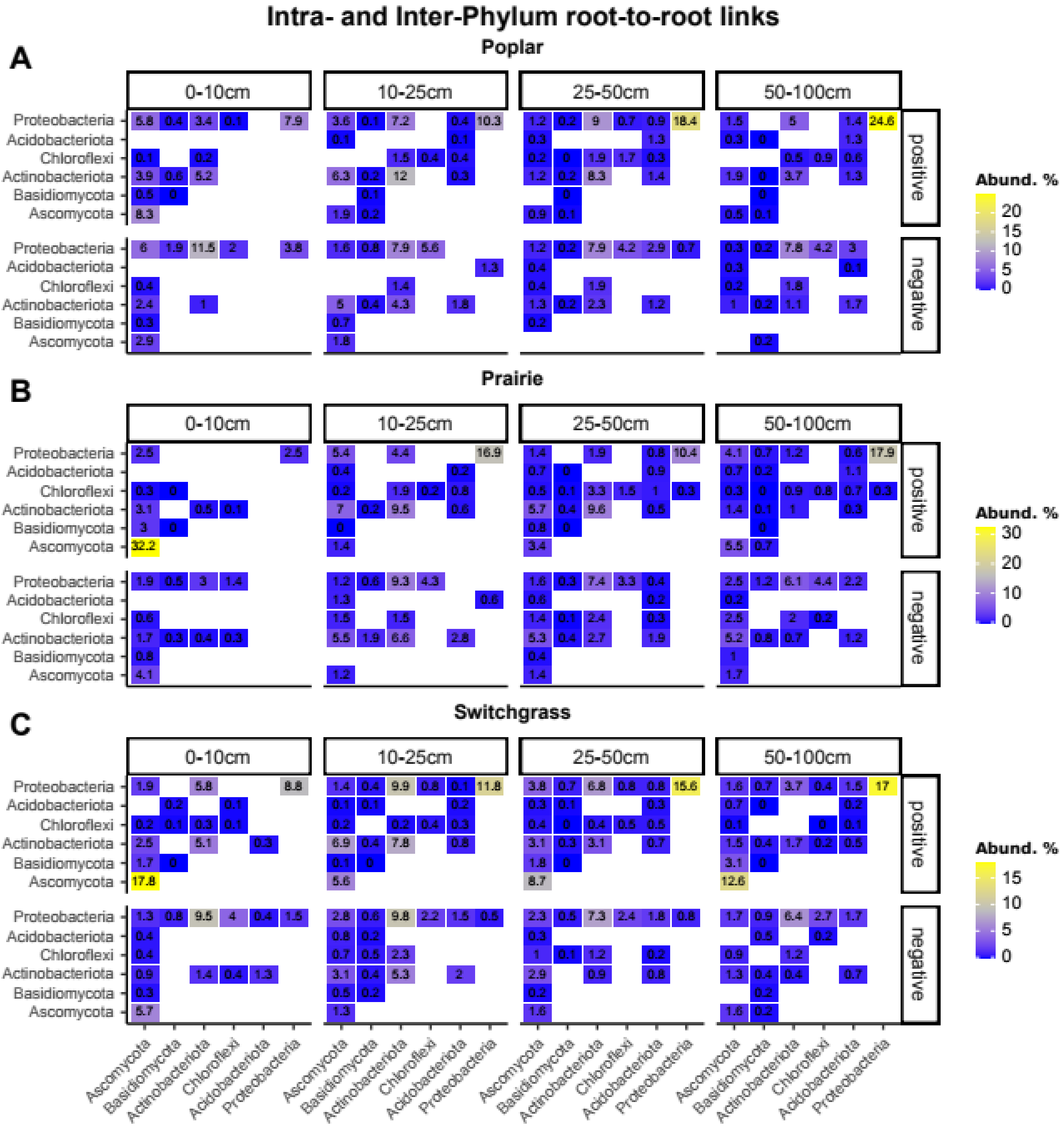

Table S1 Richness and Shannon index of root and soil samples across depth gradient for fungal and bacterial communities. Differences were tested using ANOVA or Kriskal Wallis tests where the dataset did not meet normality and/or homoscedasticity prerequisites. Signif. Codes: “0.001” (***), “0.01” (**), “ ” (not significant).

		Poplar		Prairie	
	**Factor**	**Richness**	**Shannon index**	**Richness**	**Shannon index**
**Fungi**	*Niche*	$445.98(_{1, 110)}^{***}$	$15.10_{(1, 110)}^{***}$	$132.76(_{1, 105)}^{***}$	$53.23_{(1, 105)}^{***}$
	Depth	$71.98_{(3, 110)}^{***}$	$4.49_{(3, 110)}^{**}$	$81.01_{(3, 105)}^{***}$	$6.62_{(3, 105)}^{***}$
	Niche:Depth	$85.13_{(3, 110)}^{***}$	$1.93_{(3, 110)}$	$31.13_{(3, 105)}^{***}$	$1.64_{(3, 109)}$
**Prokaryotes**	*Niche*	$270.72_{(1, 109)}^{***}$	$28.25_{(1, 109)}^{***}$	$68.46(_{1, 110)}^{***}$	$30.84_{(1, 110)}^{***}$
	Depth	$49.31_{(3, 109)}^{***}$	$3.675_{(3, 109)}^{*}$	$27.73_{(3, 110)}^{***}$	$0.46_{(3, 110)}$
	Niche:Depth	$76.21_{(3, 109)}^{***}$	$0.56_{(3, 109)}$	$55.23_{(3, 110)}^{***}$	$1.91_{(3, 110)}$

Table S2 List of all the hub taxa (highly connected, e.g. have many links, OTUs across the whole network) present in the networks. All OTUs were module hubs (OTUs more densely connected to OTUs within a module, compared to other OTUs in the network) beside Rhizobacter (see note 1). To identify hubs we used the ratio between within-module (Zi) and between-module connectivity (Pi): network hubs (Zi > 2.5, Pi > 0.62), module hubs (Zi > 2.5, Pi ≤ 0.62), connectors (Zi ≤ 2.5, Pi > 0.62) or peripherals (Zi ≤ 2.5, Pi ≤ 0.62). NA = not assigned to a niche.

OTU_ID	Taxonomy	Crop	Depth (cm)	Abundance in root	Niche	Neutral fit	Degree	Betweenness
FOTU_550	*Operculomyces* sp.	Poplar	0-10	1.68	NA	Above	9	656.22
POTU_12	*Kineosporia* sp.	Poplar	0-10	91.75	Root	Neutral	11	565.59
POTU_22	*Gaiella* sp.	Poplar	25-50	19.76	Soil	Above	9	666.68
POTU_37	Gemmatimonadetes	Poplar	25-50	2.03	Soil	Neutral	14	876.38
POTU_26	*Rhizobacter* sp.	Poplar	25-50	96.83	Root	Neutral	23	2456.14
POTU_6310	Actinobacteria	Poplar	25-50	5.05	Soil	Below	18	1721.41
POTU_8	*Actinocorallia* sp.	Poplar	50-100	97.17	Root	Neutral	13	757.57
POTU_26	*Rhizobacter* sp.	Poplar	50-100	93.54	Root	Above	28	2081.37
POTU_1451	Chloroflexi	Poplar	50-100	1.07	Soil	Below	14	1135.34
POTU_117	*Chryseolinea* sp.	Poplar	50-100	98.92	Root	Below	12	492.52
POTU_26	*Rhizobacter* sp.	Poplar	0-10	87.31	Root	Neutral	31	3451.95
FOTU_551	Glomeromycotina	Prairie	0-10	88.96	NA	Neutral	13	1238.71
POTU_5	Chloroflexi	Prairie	0-10	11.09	Soil	Neutral	21	2953.49
POTU_26	*Rhizobacter* sp. [1]	Prairie	10-25	91.84	Root	Neutral	13	845.98
POTU_35	*Actinoplanes* sp.	Prairie	25-50	97.01	Root	Neutral	16	1013.25
POTU_95	*Pseudonocardia* sp.	Prairie	25-50	44.71	Soil	Neutral	14	1450.39
POTU_2	*Bradyrhizobium* sp.	Prairie	50-100	95.97	Root	Neutral	20	1309.48
POTU_131	*Gaiella* sp.	Prairie	50-100	11.85	Soil	Neutral	10	563.47
POTU_752	Chloroflexi	Prairie	50-100	6.39	Soil	Neutral	14	1054.59
POTU_26	*Rhizobacter* sp.	Prairie	50-100	96.28	Root	Above	22	1623.3
POTU_98	Candidatus udaeobacter sp.	Prairie	50-100	10.92	Soil	Above	15	769.81
FOTU_292	*Cyphellophora* sp.	Switchgrass	0-10	84.64	NA	Above	14	971.05
POTU_15	*Acidibacter* sp.	Switchgrass	0-10	95.55	Root	Neutral	10	526.27
FOTU_8	*Acremonium* sp.	Switchgrass	10-25	0.52	Soil	Below	15	1408.51
POTU_111	Rokubacteriales	Switchgrass	10-25	15.87	Soil	Above	13	927.1
POTU_22	Gaiella sp.	Switchgrass	10-25	10.61	Soil	Neutral	19	1542.64
POTU_35	Micromonosporaceae	Switchgrass	10-25	92.64	Root	Neutral	13	1092.88
POTU_39	*Devosia* sp.	Switchgrass	10-25	90.42	Root	Neutral	17	723.22
FOTU_8	*Acremonium* sp.	Switchgrass	25-50	2.17	Soil	Above	25	4968.24
FOTU_25	Mucoromycotina	Switchgrass	25-50	0.26	Soil	Below	12	1402.67
FOTU_51	*Cladosporium* sp.	Switchgrass	25-50	27.44	NA	Above	11	861.98
POTU_22	*Gaiella* sp.	Switchgrass	25-50	10.74	Soil	Below	27	5148.61
POTU_18	Acidobacteriota	Switchgrass	25-50	18.3	Soil	Neutral	21	4427.77
FOTU_8	*Acremonium* sp.	Switchgrass	50-100	1.25	Soil	Neutral	17	1264.05
FOTU_1137	*Pezicula* sp.	Switchgrass	50-100	83.13	NA	Above	10	1663.26
FOTU_1446	Helotiales	Switchgrass	50-100	93.02	NA	Neutral	12	1894.16
POTU_111	Rokubacteriales	Switchgrass	50-100	8.87	Soil	Above	16	972.58
POTU_352	*Nitrospira* sp.	Switchgrass	50-100	1.93	Soil	Below	13	1075.99

(1) *Rhizobacter* was the only network hub, all others were module hubs.

Table S3 Microbial networks properties. Network properties that were able to best predict differences across depths in all crops (Random Forest feature selection method) are highlighted in bold.

**Network properties**	**Poplar**				**Prairie**				**Switchgrass**			
	**0-10cm**	**10-25cm**	**25-50cm**	**50-100cm**	**0-10cm**	**10-25cm**	**25-50cm**	**50-100cm**	**0-10cm**	**10-25cm**	**25-50cm**	**50-100cm**
Nodes	197	161	216	193	219	110	252	174	152	233	360	234
links	363	267	509	440	405	155	667	337	266	548	1159	563
Positive links	237	159	344	295	328	93	420	193	185	350	810	390
**Negative links**	126	108	165	145	77	62	247	144	81	198	349	173
Positive/negative links	1.881	1.472	2.085	2.034	4.26	1.5	1.7	1.34	2.284	1.768	2.321	2.254
Core OTUs – Above	50.761	34.783	21.296	24.87	44.292	14.545	38.889	48.276	32.237	24.464	43.056	20.513
Core OTUs – Neutral	41.624	55.901	61.574	45.596	42.922	67.273	54.762	46.552	51.974	65.236	42.5	56.838
Core OTUs – Below	7.614	9.317	17.13	29.534	12.785	18.182	6.349	5.172	15.789	10.3	14.444	22.65
All OTUs – Above	7.83	7.281	7.113	6.671	7.093	8.199	6.607	6.478	8.21	7.086	7.982	6.101
All OTUs – Above	87.55	88.703	88.634	89.06	88.366	87.928	89.184	89.074	87.856	89.358	87.692	89.056
All OTUs – Below	4.62	4.016	4.253	4.27	4.542	3.873	4.209	4.448	3.934	3.556	4.326	4.842
Transitivity	0.195	0.208	0.171	0.264	0.197	0.287	0.182	0.224	0.226	0.201	0.118	0.185
**Modularity**	0.526	0.487	0.524	0.546	0.593	0.569	0.481	0.527	0.542	0.495	0.495	0.55
Modules	42	49	29	33	39	35	32	36	34	33	14	31
**Average module size**	4.69	3.286	7.448	5.848	5.615	3.143	7.875	4.833	4.471	7.061	25.714	7.548
Module SD	8.348	5.53	12.24	11.525	9.138	4.797	14.712	9.016	7.597	12.715	37.36	14.137
**Average degree**	3.685	3.317	4.713	4.56	3.699	2.818	5.294	3.874	3.5	4.704	6.439	4.812
Degree SD	3.902	3.642	3.83	4.008	3.185	2.903	4.167	3.644	3.428	3.988	4.218	3.74
Average betweenes	182.716	109.745	240.435	189.964	256.909	83.564	277.972	170.695	143.612	272.026	477.608	276.722
Betweenness SD	346.195	239.039	333.256	307.191	407.345	152.292	382.997	276.24	279.654	350.524	596.318	349.518
Path length	3.794	3.559	3.718	3.742	4.304	3.907	3.683	3.768	3.862	3.916	3.752	3.95
Edge density	0.019	0.021	0.022	0.024	0.017	0.026	0.021	0.022	0.023	0.02	0.018	0.021
Network diameter	10	8	9	8	10	8	10	11	9	10	10	9
**Module hubs**	2	0	4	4	2	1	2	5	2	5	5	5
Network hubs	1	0	0	0	0	0	0	0	0	0	0	0
Conncetors	19	19	24	11	17	5	37	18	11	25	32	12
Peripherals	175	142	188	178	200	104	211	151	139	203	323	217
Fungal OTUs	144	75	55	51	195	47	122	96	93	110	213	139
Bacterial OTUs	53	85	159	140	24	63	129	78	59	122	147	94
Archaeal OTUs	0	1	2	2	0	0	1	0	0	1	0	1
Positive interkingdom OTUs	49	41	53	42	45	26	89	46	28	80	157	78
Negative interkingdom OTUs	46	31	28	18	25	21	88	56	11	56	96	43
Root indicators	47	49	62	63	31	43	61	40	47	67	88	71
Soil indicators	40	65	107	91	36	36	94	59	36	79	90	53
Poplar indicators	7	9	6	5	2	2	5	3	3	3	5	1
Prairie indicators	1	1	2	1	3	3	3	3	1	3	3	3
Switchgrass indicators	2	1	1	2	2	1	4	3	7	8	10	11
10cm indicators	8	2	2	0	7	1	4	3	8	3	4	1
25cm indicators	2	2	1	1	2	1	2	1	1	2	2	0
50cm indicators	0	0	0	0	0	0	0	0	0	0	0	0
100cm indicators	3	3	4	9	1	1	5	6	1	2	5	13
